# Different genetic structures revealed resident populations of a specialist parasitoid wasp in contrast to its migratory host

**DOI:** 10.1002/ece3.3097

**Published:** 2017-06-12

**Authors:** Shu‐Jun Wei, Yuan Zhou, Xu‐Lei Fan, Ary A. Hoffmann, Li‐Jun Cao, Xue‐Xin Chen, Zai‐Fu Xu

**Affiliations:** ^1^ Institute of Plant and Environmental Protection Beijing Academy of Agriculture and Forestry Sciences Beijing China; ^2^ College of Agriculture South China Agricultural University Guangzhou China; ^3^ School of BioSciences Bio21 Institute The University of Melbourne Parkville VIC Australia; ^4^ Institute of Insect Sciences Zhejiang University Hangzhou China

**Keywords:** *Cotesia vestalis*, dispersal, host–parasitoid interaction, migration, *Plutella xylostella*, population genetic structure

## Abstract

Genetic comparisons of parasitoids and their hosts are expected to reflect ecological and evolutionary processes that influence the interactions between species. The parasitoid wasp, *Cotesia vestalis,* and its host diamondback moth (DBM), *Plutella xylostella,* provide opportunities to test whether the specialist natural enemy migrates seasonally with its host or occurs as resident population. We genotyped 17 microsatellite loci and two mitochondrial genes for 158 female adults of *C. vestalis* collected from 12 geographical populations, as well as nine microsatellite loci for 127 DBM larvae from six separate sites. The samplings covered both the likely source (southern) and immigrant (northern) areas of DBM from China. Populations of *C. vestalis* fell into three groups, pointing to isolation in northwestern and southwestern China and strong genetic differentiation of these populations from others in central and eastern China. In contrast, DBM showed much weaker genetic differentiation and high rates of gene flow. TESS analysis identified the immigrant populations of DBM as showing admixture in northern China. Genetic disconnect between *C. vestalis* and its host suggests that the parasitoid did not migrate yearly with its host but likely consisted of resident populations in places where its host could not survive in winter.

## 1 INTRODUCTION

Parasitism is one of the most important biological interactions that shapes biodiversity and influences community processes (Hudson, Dobson, & Lafferty, 2006; Wood et al., 2007). Herbivorous insects and their parasitoids constitute species‐rich communities in terrestrial ecosystems (May & Beverton, 1990). Parasitoid larvae feed on and ultimately cause the death of their hosts, leading to hosts evolving mechanisms to evade parasitoids and reduce parasitism (Shiojiri, Takabayashi, Yano, & Takafuji, 2002). This is countered by strong selection in parasitoids to find and attack their hosts (Jervis, Ellers, & Harvey, 2008). The ecological interactions between hosts and parasitoids can be influenced by complicated factors (Godfray, 1994; van Nouhuys, 2016).

Comparisons on population genetic structure of herbivorous insects and their parasitoids in space and time provide new ways to reveal the ecological and evolutionary processes that influence the interactions between species (Crutsinger, 2016; Stone et al., 2012; Sutton, Riegler, & Cook, 2016). Current studies on population genetic structure have showed that parasitoids can track hosts (Gebiola, Lopez‐Vaamonde, Nappo, & Bernardo, 2014; Hayward & Stone, 2006; Stone et al., 2012), shift to different hosts (Gebiola et al., 2014; Nicholls et al., 2010), and locate hosts in fragmented landscapes (Couchoux, Seppa, & van Nouhuys, 2016; Hayward & Stone, 2006; Nair, Fountain, Ikonen, Ojanen, & van Nouhuys, 2016; Sutton et al., 2016).

Seasonal migration of herbivorous insects to new habitats, which provides “enemy‐free space”, has been considered as one of the avoidance behaviors that have evolved in the host in response to parasitism (Chapman, Reynolds, & Wilson, 2015). However, parasitoids may evolve to synchronize migration with their hosts (Pérez‐Rodríguez, Shortall, & Bell, 2015), and even ahead of their hosts (Bortolotto, Júnior, & Hoshino, 2015). For instance, some Aphidiinae parasitoids show long‐range dispersal in egg and larval stages through host flight as well as through active dispersal by adult parasitoids (Bortolotto et al., 2015; Derocles, Plantegenest, Chaubet, Dedryver, & Le Ralec, 2014). Migratory pests arriving at a location might also be attacked by resident parasitoids (Tanaka, Nishida, & Ohsaki, 2007). However, there is little information on the population genetic structure of migratory hosts and their specialist parasitoids.

The diamondback moth (DBM), *Plutella xylostella* (L.) (Lepidoptera: Plutellidae), is one of the most destructive pests of cruciferous plants worldwide (Furlong, Wright, & Dosdall, 2013; Rincon, Bordat, Löhr, & Dupas, 2006). Studies have shown that DBM is a migratory species (Endersby, McKechnie, Ridland, & Weeks, 2006; Furlong et al., 2013). In northern China, DBM cannot survive low winter temperatures, and northern areas appear to be invaded annually from southern regions by long‐distance migration, with only a low level of reverse migration likely (Fu, Xing, Liu, Ali, & Wu, 2014; Wei et al., 2013; Yang et al., 2015). The parasitoid wasp, *Cotesia vestalis* Haliday (Hymenoptera: Braconidae), is an important natural enemy of DBM (Furlong et al., 2013). Although *C. vestalis* can attack and develop in other lepidopteran species in the laboratory (Cameron, Walker, Keller, & Clearwater, 1997), it is generally regarded as a specialist parasitoid of DBM (Arvanitakis, David, & Bordat, 2014). This parasitoid appears to be as widespread as DBM and occurs in its migratory range (Furlong et al., 2013; Grzywacz et al., 2010). The similar distribution of *C. vestalis* and its host, and the host‐specific relationship between the parasitoid and DBM, make this an interesting system for comparing population genetic structure across trophic levels.

Natural populations of *C. vestalis* in high latitude regions could be induced to diapause at the prepupal stage, while those in low latitude region did not show develop arrest in all or most of the individuals (Ahmed et al., 2007; Alvi & Momoi, 1994). This may reflect local adaptation to specific temperature and photoperiod conditions. However, it is possible that high levels of gene flow are present in this species, given that populations of *C. vestalis* in temperate regions are thought to include migratory individuals as well as those emerging from diapause (Furlong et al., 2013). It is therefore not clear whether *C. vestalis* migrates mostly along with DBM, resulting in high levels of gene flow between populations, or if the parasitoid is largely resident.

In this study, we compare the genetic structure of *C. vestalis* from different regions of China with that of its host species, DBM, based on analyses of microsatellite loci and mitochondrial genes. We predict that whether *C. vestalis* consists mostly of resident populations, it should show different genetic structure from DBM; however, if the *C. vestalis* migrate seasonally following its host, both species tend to show weak genetic structure among geographical populations due to the high level of gene flow. The results of our study will help to illustrate the processes that influence the interactions between a specialist parasitoid and its migratory host and guide the conservation and utilization of *C. vestalis* for pest control, more generally.

## 2 MATERIALS AND METHODS

### 2.1 Sample collection and DNA extraction

**Figure 1:**
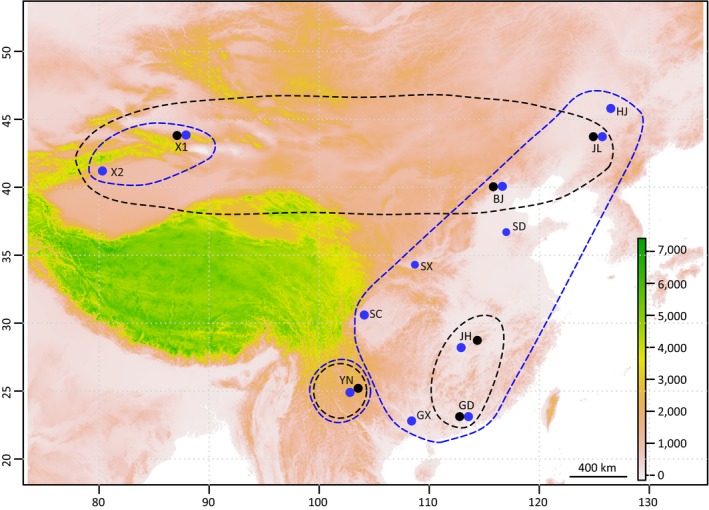
Collection sites and genetic groups of *Cotesia vestalis* and its host *Plutella xylostella*. The blue points indicate the collection sites for *C. vestalis* while the black points indicate the collection sites for *P. xylostella*. Three genetic groups of *C. vestalis* (blue circles), that is, XJ (Xingjiang group includes X1 and X2), CE (central and east group includes HJ, JL, BJ, SX, SD, JH, GD, GX, and SC), and YN (Yunnan group), and three groups of *P. xylostella* (black circles), *that is,* NT (northern group includes X1, JL, and BJ), ST (southern group includes JH and GD), and YN (Yunnan group), inferred from population genetic structure analyses are shown on the map. Codes for the populations are presented in Table 1

DBM larvae were collected from cabbage fields and reared in the laboratory at room temperature until *C. vestalis* cocoons or adults were evident. In total, 158 female adults of *C. vestalis* were collected from 12 geographically separate sites, and 127 DBM larvae were collected from six separate sites across China (Table 1 and Figure 1). Female adults of *C. vestalis* were used for genotyping rather than males because of haplodiploidy in this wasp. Genomic DNA was extracted from single specimen using the DNeasy Blood and Tissue Kit (Qiagen, Germany).

**Table 1:** Sample collection information of *Cotesia vestalis* and its host *Plutella xylostella* used in this study

Population code	Collection locality	Latitude (N)	Longitude (E)	Number of CV	Number of DBM
X1	Urumqi of Xinjiang Uygur Autonomous Region	43°49′31	87°36′50	6	15
X2	Aral of Xinjiang Uygur Autonomous Region	41°09′59	80°15′40	14	–
HJ	Ha'erbin of Heilongjiang Province	45°48′00	126°31′47	24	–
JL	Changchun of Jilin Province	43°48′48	125°19′01	10	24
BJ	Haidian district of Beijing	39°57′28	116°17′32	13	24
SD	Shanghe of Shandong Province	36°40′34	117°00′45	9	–
SX	Yangling of Shaanxi Province	34°19′50	108°42′16	10	–
SC	Chengdu of Sichuan Province	30°34′21	104°03′44	6	–
JH	Nanchang of Jiangxi Province	28°41′07	115°51′10″	–	24
	Changsha of Hunan Province	28°13′52	112°56′00	12	–
GD	Guangzhou of Guangdong Province	23°07′54	113°15′32	16	24
GX	Nanning of Guangxi Zhuang Autonomous Region	22°49′09	108°21′46	18	–
YN	Kunming of Yunnan Province	24°52′57	102°49′54	20	–
	Qujing of Yunnan Province	25°29′35	103°47′39	–	16

CV, *Cotesia vestalis*; DBM, diamondback moth *Plutella xylostella*. “–” indicates no collection of specimens from the corresponding locality.

### 2.2 Microsatellite genotyping

*Cotesia vestalis* were genotyped at 17 microsatellite loci developed from the genome of *C. vestalis* (Appendix S1), while DBM was genotyped at nine microsatellite loci developed by Esselink, Den Belder, Elderson, and Smulders (2006) as used in Wei et al. (2013). In order to improve efficiency and lower cost for microsatellite amplification, we used a method of adding a primer tail C to the 5′ end of the candidate forward primer (Blacket, Robin, Good, Lee, & Miller, 2012). The size of the amplified PCR products was determined using an ABI 3730 xl DNA Analyzer with a GeneScan 500 LIZ size standard.

### 2.3 Genetic diversity analysis

The microsatellite loci determined from GENEMAPPER were checked for stuttering and large allele dropout by MICROCHECKER version 2.2.3 (van Oosterhout, Hutchinson, Wills, & Shipley, 2004). Null allele frequencies at each locus were calculated using the R package GENELAND version 4.0.4 (Guillot, Mortier, & Estoup, 2005). Tests for linkage disequilibrium (LD) and deviations from Hardy–Weinberg equilibrium (HWE) at each locus and each population were conducted with GENEPOP version 4.2.1 (Rousset, 2008). Genetic differentiation (F_ST_) as well as observed and expected heterozygosities were calculated using MSA version 4.0.5 (Dieringer & Schlötterer, 2003). Estimates of inbreeding, number of alleles and allelic richness were obtained using FSTAT version 2.9.3 (Goudet, 1995).

### 2.4 Population genetic structure

We performed Mantel tests of genetic distance versus geographical distance across populations using the R package ade4 with 10,000 permutations. Pairwise F_ST_ was calculated using GENEPOP version 4.2.1 (Rousset, 2008).

Two Bayesian clustering methods were used to seek the most likely population structure based on the microsatellite loci. First, the genetic structure among populations and individuals over the microsatellite data set was investigated for each species using STRUCTURE version 2.3.1. (Pritchard, Stephens, & Donnelly, 2000). Thirty independent runs were performed, setting the number of clusters (*K*) from 1 to 10. Each run started with a burn‐in of 100,000 followed by 1,000,000 iterations employing the admixture model. The final true value of *K* was determined using the web software STRUCTURE HARVESTER version 0.6.94 (Earl & vonHoldt, 2011) and then using the highest value of ΔK in the likelihood distribution. The results were permuted with CLUMPP version 1.1.2 (Jakobsson & Rosenberg, 2007), and the mean of the permuted results was plotted by using DISTRUCT version 1.1 (Rosenberg, 2003).

Second, TESS version 2.3.1 (Chen, Durand, Forbes, & Francois, 2007), which implements a conditional autoregressive Gaussian model and an admixture model, was used to estimate spatially varying individual admixture proportions. We ran 10 replicates for each *K* value (from 2 to 10) with 120,000 MCMC sweeps after a burn‐in of 2,000. The optimal *K* value was chosen by inspecting bar plots of individual membership probabilities and the *K* value at which the plots of Deviance Information Criterion (DIC) values against *K* were stable across replicates and began to plateau. The optimal *K* value was replicated 100 times, and 20% of the replicates with the lowest DIC were processed with CLUMPP version 1.1.2 (Jakobsson & Rosenberg, 2007).

### 2.5 Gene flow analysis

We estimated asymmetric gene flow between populations using the software MIGRATE version 3.6.11 (Beerli & Felsenstein, 2001) based on microsatellite loci. The parameters used are as follows: long‐chains = 1, long‐inc = 500, long‐sample = 2,000, burn‐in = 10,000, heating = YES: 1: (1.0, 1.5, 3.0, 6.0), heated‐swap = NO and replicate = YES: 5. Four runs of MIGRATE analysis were conducted to confirm consistency of the patterns. For each run, we changed the random number seed and starting values for θ and *M*. In the first run, θ and *M* were estimated from F_ST_ values. In the subsequent runs, Bayesian estimates of θ and *M* from the previous run were used.

### 2.6 Mitochondrial gene sequencing and analysis in *C. vestalis*

In order to validate morphological identification of *C. vestalis*, two mitochondrial gene segments from the cytochrome oxidase subunit I gene (*cox1*, 624 bp) and the mitochondrial cytochrome b gene (*cob*, 879 bp) were sequenced. All PCRs were conducted using the Mastercycler pro system (Eppendorf, Germany) following standard PCR amplifications conditions and an annealing temperature of 55°C. Amplified products were purified and sequenced directly from both strands using an ABI 3730 xl DNA Analyzer (Applied Biosystems, USA).

The sequencing results determined from both strands were assembled. Each gene was aligned independently with CLUSTALW (Thompson, Higgins, & Gibson, 1994) implemented in MEGA version 5 (Tamura et al., 2011) using the default set of parameters. Alignment of the nucleotide sequences of the protein‐coding genes was guided by amino acid alignment. The standard diversity indices and background selection using Tajima's D were calculated with ARLEQUIN version 3.5 (Excoffier & Lischer, 2010). The pairwise distance was calculated in MEGA version 5 (Tamura et al., 2011) with the K2P model.

The split network was constructed using SPLITSTREE version 4.13.1 (Huson & Bryant, 2006) from combined mitochondrial genes to reveal relationships among haplotypes. The neighbor‐net method was used for construction of networks under a distance model of K2P. The statistically significant split with >95% confidence was identified after 1,000 bootstraps.

## 3 RESULTS

### 3.1 Genetic diversity

For the microsatellite loci characterized in both species, null allele frequencies are low. No locus was significantly linked across all populations. No locus departed from HWE in all populations and no population departed from HWE across all loci. Both species showed high allelic richness, but allelic richness of DBM (7.4–8.9) was somewhat greater than that of *C. vestalis* (3.1–3.8).

### 3.2 Population genetic structure

The level of pairwise population differentiation is strong in *C. vestalis*, but weak in DBM (Table 2). The Mantel tests indicated a correlation between genetic and geographical distance in both species with an r value of 0.643 (*p* = .005) for *C. vestalis* and an r value of 0.579 (*p* = .046) for DBM.

**Table 2:** Pairwise F_ST_ among populations of *Cotesia vestalis* and its host *Plutella xylostella* based on microsatellite loci

	Population	HJ	JLC	SX	BJH	SD	JH	GD	GX	YN	SC	XJ
*Cotesia vestalis*	JL	0.028^a^										
	SX	0.005	0.051^a^									
	BJ	0.020^a^	0.063^a^	0.017								
	SD	0.000	0.028	−0.006	0.013							
	JH	0.004	0.049^a^	0.018	0.023^a^	0.000						
	GD	0.059^a^	0.079^a^	0.073^a^	0.072^a^	0.073^a^	0.044^a^					
	GX	0.031^a^	0.070^a^	0.037^a^	0.045^a^	0.016	0.030^a^	0.049^a^				
	YN	0.061^a^	0.103^a^	0.060^a^	0.077^a^	0.059^a^	0.077^a^	0.135^a^	0.081^a^			
	SC	0.024	0.064^a^	0.019	0.028	0.004	0.030	0.077^a^	0.033	0.061^a^		
	X2	0.133^a^	0.157^a^	0.152^a^	0.181^a^	0.146^a^	0.158^a^	0.215^a^	0.172^a^	0.176^a^	0.171^a^	
	X1	0.166^a^	0.213^a^	0.200^a^	0.217^a^	0.200^a^	0.215^a^	0.286^a^	0.220^a^	0.211^a^	0.212^a^	0.088^a^

	Population	X1	JL	BJ	JH	GD
*Plutella xylostella*	JL	0.022^a^				
	BJ	0.025^a^	0.008^a^			
	JH	0.038^a^	0.017^a^	0.012^a^		
	GD	0.064^a^	0.036^a^	0.022^a^	0.019^a^	
	YN	0.030^a^	0.046^a^	0.035^a^	0.047^a^	0.055^a^

a Indicates significant difference at *p* = .05.

**Figure 2:**
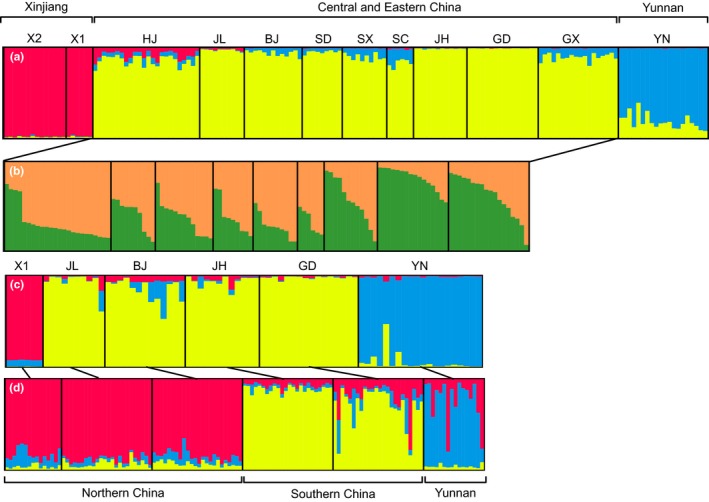
Membership coefficients of *Cotesia vestalis* (a–c) and it host *Plutella xylostella* (d) estimated by the Bayesian individual clustering method implemented in STRUCTURE based on microsatellite loci. For the *C. vestalis*, all populations (a), the central and eastern populations (b) and six representative populations with data for both species (c) were separately analyzed. In *Cotesia vestalis*, two populations from Xinjiang of northwestern China formed one cluster (named Xinjiang, red), while in *P. xylostella*, all three northern populations formed one cluster (named Northern China in red, figure d). Each vertical line represents an individual, and each colour represents a genetic cluster. Both species had the Yunnan cluster from the Yunnan province. Codes of the population name are as in Table 1

The structure analysis of *C. vestalis* showed that northwestern populations from Xinjiang formed one cluster, while the southwestern population from Yunnan formed another distinct cluster (Figure 2a). When these three populations were excluded for analysis, two clusters were identified along with a north–south gradient of populations with increasing contributions from one of the two clusters (Figure 2b). When the analysis was conducted using only the locations with sampling for both *C. vestalis* and DBM, the same genetic structure was generated (Figure 2c). In DBM, three clusters were identified. The first cluster was formed by the three northern populations, the second one was formed by the two southern populations, and the third was formed by the southwestern population. The proportion of admixture was higher in DBM populations than in *C. vestalis* populations (Figure 2d).

**Figure 3:**
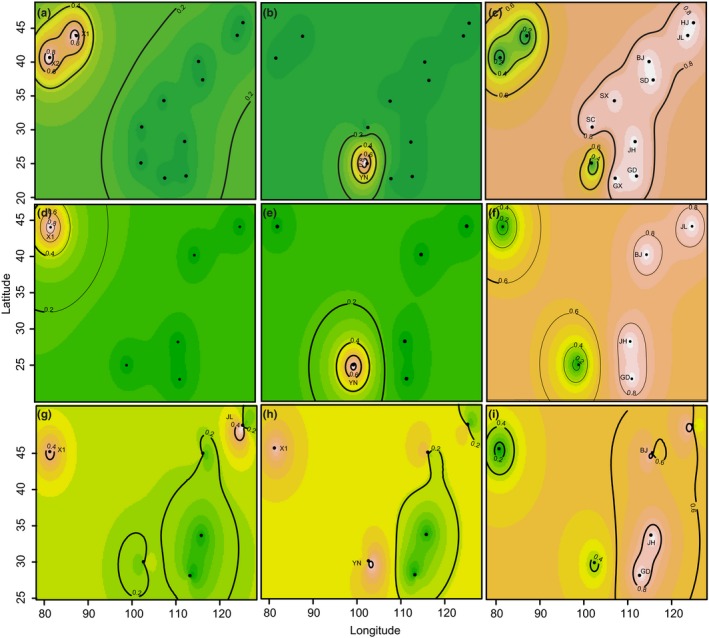
Population genetic structure of *Cotesia vestalis* and its host *Plutella xylostella* inferred by TESS based on microsatellite loci. Higher probabilities of population membership are illustrated in lighter shading. Figures a, b, and c show the three inferred clusters of *C. vestalis* when all populations were included, Figures d, e, f show the three inferred clusters of *C. vestalis* when six populations with data for both species were included, and figures g, h, i show the three inferred clusters of *P. xylostella*

With the TESS analysis, admixture patterns of populations of DBM were identified. All populations of *C. vestalis* were assigned to their three corresponding clusters with high probabilities (>0.8); while in DBM, an admixture zone was identified in northern populations. The northwestern population was similar to another northern population and the southwestern population, while the other two northern populations were found to be an admixture of southern populations, respectively (Figure 3).

### 3.3 Gene flow estimates

The migration (*m*,* N*_*e*_ *m*) estimates obtained from the microsatellite data of *C. vestalis* show low levels of effective migrants per generation between each pair of the three groups (0.01–52.43) and between the individual population pairs (0.65–17.77). In contrast, estimates of 29.2–249.72 were obtained for the clusters of DBM and estimates of 4.88–91.93 for population pairs of DBM. These estimates indicate higher movement rates for the host in comparison with its parasitoid.

### 3.4 Genetic diversity and phylogenetic networks of *C. vestalis* based on mitochondrial genes

In *C. vestalis*, 12 mitochondrial haplotypes were identified from the combined mitochondrial genes. The southwestern population from Yunnan had both high nucleotide diversity and haplotype diversity. Tajima's D calculated for each of the 12 populations showed that only two populations significantly deviated from neutrality.

**Figure 4:**
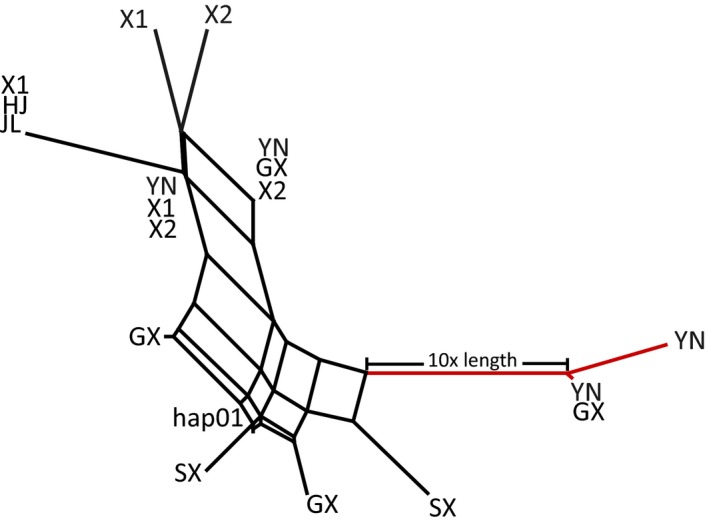
The splits tree analysis of the haplotypes for *C. vestalis* based on the combined mitochondrial genes. Population codes in which the haplotype present are indicated. Hap01 is present in HJ, JL, BJ, SD, JH, SX, SC, GD, GX, and YN. The two red haplotypes are distantly related to others. Population codes reflecting origins of the haplotypes are given in Table 1

The SPLITSTREE analysis revealed two haplotype lineages: A minor one, which contained most haplotypes of partial YN and GX populations (the southern region of China), and a major one, which consisted of other haplotypes of all populations (Figure 4). No significant signature of reticulation (with >95% confidence) was detected. The pairwise distance between the haplotypes from the major lineage and that from the minor lineage varied from 0.014 to 0.017, while that between haplotypes within each lineage varied from 0.001 to 0.004.

## 4 DISCUSSION

### 4.1 Comparison of population structure

This study examined the population structure of *C. vestalis* and its host DBM. We collected specimens from southern regions of China extending to the northern regions, covering both emigrant (southern) and immigrant (northern) areas of DBM (Figure 1) (Furlong et al., 2013). Our previous work examined population genetic structure of DBM across China by dense sampling of 27 populations (Wei et al., 2013). In this study, we selected five populations of DBM from Wei et al. (2013) and one newly collected population from Xinjiang to fill a gap in northwestern China. Because the population genetic structure of *C. vestalis* was first examined in China, we included more population for this species than DBM. The sampling difference between host and parasitoid did not influence the analyses of population structure, as evident in the subset analysis using only the locations with data for both species (Figures 2 and 3).

In *C. vestalis*, populations from Xinjiang in northwestern China and Yunnan province in southwestern China formed two groups. The differentiation between two northwestern populations was also high, which might be caused by the isolation of Tianshan Mountains (average altitude is about 5,000 m). Although *C. vestalis* may show low levels of genetic differentiation across fields as has been found within Taiwan, indicative of high gene flow in *C. vestalis* at a local scale (de Boer, Groenen, Pannebakker, Beukeboom, & Kraus, 2015), across distances of several hundred kilometers migration appears less likely. The observations we have made here are also consistent with evidence for structured populations and reproductive isolation of *C. vestalis* in Japan and Myanmar (Htwe, Takagi, & Takasu, 2009).

In contrast, DBM showed a different population structure to its parasitoid. Earlier work on DBM indicated no strong correlation between genetic and geographical distance among populations in China (Wei et al., 2013; Yang et al., 2015), which contrasts with the situation in *C. vestalis*. Although DBM is a migratory species, we found weak genetic structure among populations in its native range as reported in our previous study (Wei et al., 2015). As no point‐to‐point migration occurred in DBM from southern to northern China, immigrant populations in northern regions might be composed of different populations from southern regions. This was suggested by the presence of admixture in northern populations identified by a TESS analysis. The routes of migration from south to north, with little reverse migration in China (Wei et al., 2015), mark the three mixed populations in northern China as one large group.

### 4.2 Resident populations of *Cotesia vestalis*

Estimates of gene flow for *C. vestalis* were low, in sharp contrast to that of its DBM host. The high rates of gene flow for DBM are consistent with estimates for this pest obtained from other studies (Wei et al., 2015; Yang et al., 2015). Strong population structure and limited gene flow are likely in *C. vestalis* resident in the Xinjiang area, in contrast to species which migrate from southern to northern China (Fu et al., 2014; Wei et al., 2013). Biological observations on *C. vestalis* point to the potential for residency in this species. When the second and third larval instars of *C. vestalis* are subjected to temperatures below 17°C and 13‐hr light per day, progeny may be induced to enter facultative diapause in the prepupal stage outside the host (Ahmed et al., 2007; Alvi & Momoi, 1994). This may help *C. vestalis* to overwinter in regions where DBM could not survive, as in northern Japan and North Korea (Alvi & Momoi, 1994). It is unlikely that Xinjiang populations are immigrants from other regions in China, as genetic differentiation between the two Xinjiang populations was larger than that of many other pairs of populations. If *C. vestalis* migrates over long distances similar to its host, genetic differentiation would be reduced between nearby populations. Most populations showed no evidence of bottlenecks consistent with parasitoid populations having a large size (Appendix S2). This was also evident from our estimates of effective population size for the *C. vestalis* populations which varied depending on the approach used but were nevertheless similar to estimates for DBM populations (Appendix S2). This reinforces the notion that strong genetic differentiation across the parasitoid populations was a consequence of low migration levels across large‐scale regions rather than small population size.

The mitochondrial differentiation of the southwestern population from the other populations also points to genetic differentiation (Appendix S3). Twelve of 20 individuals from this population belonged to a divergent mitochondrial haplotype lineage, whereas there was only one individual with this haplotype lineage in the neighboring population GXNN, indicating limited gene flow. The presence of a divergent lineage might indicate that the region around Yunnan province is either refugial or the area of origin for *C. vestalis*, as reported for other species (Wei et al., 2015; Yang, Li, Ding, & Wang, 2008). Further studies are needed to test this idea.

In *C. vestalis,* nine populations from central and eastern China formed one large group. Nested analysis of these populations in STRUCTURE indicated two clusters whose relative importance changed from southern to northern regions, contrasting to the pattern expected of a long‐distance migratory species (Endersby et al., 2006; Lyons et al., 2012). These populations might be composed of both overwintering parasitoids and migrants. Based on field observations indicating a limited ability to spread (Lei & Camara, 1999), Quicke (2015) suggested that specialist parasitoids attacking fragmented host populations might be vulnerable to local extinction, which could in turn influence population genetic patterns (Nyabuga, Loxdale, Heckel, & Weisser, 2011). We are unaware of studies on long‐distance migration in *Cotesia*, although Lei and Camara (1999) noted limited movement ability in the related specialist parasitoid, *Cotesia melitaearum*. Based on current knowledge, it seems likely that *C. vestalis* has a limited ability to disperse over a long distance. On the other hand, cruciferous vegetables as DBM host plants are usually planted in northern China in summer and moved south as produce, while in southern China they are planted in winter and transported north as produce. This may lead to occasional long‐distance dispersal of *C. vestalis* across regions in different directions. More work is needed to trace movement patterns.

### 4.3 Risk of local extinction in *Cotesia vestalis*

Although we did not observe any overall difference in the effective population size of the parasitoid and its host, the dependency of a parasitoid on a host population may lead to a relatively smaller effective population size in some situations (Nair et al., 2016), increasing the effects of genetic drift and inbreeding, as well as the risk of local extinction (Ellstrand & Elam, 1993). For instance, local extinction and colonization events as well as inbreeding may have contributed to temporal genetic differentiation in the wasp *Lysiphlebus hirticornis* (Nyabuga et al., 2011). Local extinction may occur in northern areas also because that colonization of DBM following migration may not always be successful in the same place in successive years.

*Cotesia vestalis* has a complementary sex determination (CSD) system, in which homozygosity at CSD loci lead to the dead end of diploid males (de Boer, Ode, Vet, Whitfield, & Heimpel, 2007). This could further drive a small effective population size in *C. vestalis* with selection to avoid inbreeding as in other species (Chuine, Sauzet, Debias, & Desouhant, 2015), as proved in a field study in Taiwan island that a low level of sibling mating was found in the species (de Boer et al., 2015). Lack of genetic structure in populations collected from Taiwan island indicated that the *C. vestalis* showed dispersal ability in local area (de Boer et al., 2015), as reported in many other parasitoids (Couchoux et al., 2016; Nair et al., 2016; Nyabuga et al., 2011). The movement of *C. vestalis* in local area may explain the avoidance of local extinction of the resident populations in northern China.

### 4.4 Assembly of *Cotesia vestalis*

When hosts change their distributions, they may escape their predators (Hayward & Stone, 2006; Stone et al., 2012), but host‐parasitoid communities may re‐establish again within a relatively short time (Gebiola et al., 2014). Local populations of parasitoids may also change their host specificity and shift to attack invading hosts to which they have not recently been exposed (Gebiola et al., 2014; Nicholls et al., 2010). Some parasitoids are also able to track hosts by dispersing further and overcoming habitat fragmentation (Couchoux et al., 2016; Nair et al., 2016; Sutton et al., 2016). Our study suggests that the specialist parasitoid *C. vestalis* tracks its DBM host through local adaptation, instead of tracking DBM out of areas where the host cannot overwinter.

*Cotesia vestalis* has been recorded as attacking DBM in many regions with no records of deliberate releases (Furlong et al., 2013). Differences in diapause induction in two geographical populations of *C. vestalis* suggest life‐cycle adaptations to local environmental conditions (Ahmed et al., 2007; Alvi & Momoi, 1994). In combination with strong genetic structure among populations evident in our study, establishment of *C. vestalis* seems unlikely following a release particularly if released wasps are adapted to different conditions. Given that the wasp seems to disperse well in a local area (de Boer et al., 2015), we predict that stepping‐stone dispersal followed by adaptation to local conditions may control the dynamics and evolutionary divergence of *C. vestalis*. Host tracking of parasitoid is likely the result of “ecological sorting” (ecological resource tracking) (Ackerly, 2003), while adaptation to new environmental conditions after dispersal likely reflects “ecological fitting” (Agosta & Klemens, 2008).

## 5 CONCLUSION

We have demonstrated that the parasitoid and its host show contrasting patterns of genetic differentiation. As far as we know, this is the first comparison of genetic structure in populations of a migratory host and its specialist parasitoid in a shared landscape. Strong genetic structure revealed resident populations in the parasitoid in regions where DBM could not survive in winter. Processes of stepping‐stone dispersal followed by adaptation to local conditions may explain the current distribution of the parasitoid out the annual occurring areas of the host. Our study also provides information on conservation and usage of the parasitoid *C. vestalis* in biological control of DBM.

## CONFLICT OF INTEREST

There is no conflict of interest.

## DATA ARCHIVING STATEMENT

Data for this study are available at the Dryad Digital Repository: http://dx.doi.org/10.5061/dryad.45fm6.

## AUTHOR CONTRIBUTIONS

Shu‐Jun Wei and Zai‐Fu Xu conceived and designed the experiments; Shu‐Jun Wei and Zai‐Fu Xu organized the collection of specimens; Yuan Zhou and Xu‐Lei Fan performed the molecular analyses; Yuan Zhou, Shu‐Jun Wei, and Li‐Jun Cao analyzed the data; Shu‐Jun Wei, Yuan Zhou, Ary Hoffmann, Zai‐Fu Xu, Xue‐Xin Chen, and Li‐Jun Cao discussed the results; Shu‐Jun Wei, Yuan Zhou, Ary Hoffmann, and Zai‐Fu Xu wrote the manuscript.
