# Supplementary material for: Different genetic structures revealed resident populations of a specialist parasitoid wasp in contrast to its migratory host
Source: Ecol Evol. 2017 Jun 12;7(14):5400–9. doi: 10.1002/ece3.3097 (PMC5528221; doi:10.1002/ece3.3097)
Supplement: Supplementary file 1 [file ECE3-7-5400-s001.docx]

**Supplemental materials**

**Different genetic structures revealed resident populations of a specialist parasitoid wasp in contrast to its migratory host**

Shu-Jun Wei, Yuan Zhou, Ary Anthony Hoffmann, Xu-Lei Fan, Li-Jun Cao, Ya-Jun Gong, Xue-Xin Chen, Zai-Fu Xu

**Appendix S1** Development of microsatellite markers for *Cotesia vestalis*

**Appendix S2** Bottleneck analysis and effective population size estimation of the populations in *Cotesia vestalis* and *Plutella xylostella*

**Appendix S3** BAPS analysis of population genetic structure of *Cotesia vestalis*

**Appendix S1** Development of microsatellite markers for *Cotesia vestalis*

**Sample collection and DNA extraction**

Eight *C. vestalis* female adults collected from Changchun (Jilin Province), Beijing, Nanchang (Jiangxi Province), Fuzhou (Fujian Province), and Guangzhou (Guangdong Province) in China were used for initial evaluation of amplification efficiency of the primers and polymorphism of loci. Forty *C. vestalis* adult females from Changchun (18) and Nanchang (22) were used for further validation and evaluation of the selected microsatellite loci. Genomic DNA was extracted from single individuals using DNeasy Blood & Tissue Kit (Qiagen, Hilden, Germany).

**Microsatellite loci identification**

The genome of *C. vestalis* (Chen et al, under revision submitted to Nature) was used for microsatellite discovery. Microsatellite scanning within the genome was performed with the program SciRoKo to identify mononucleotide, dinucleotide, trinucleotide, tetranucleotide, pentanucleotide and hexanucleotide motifs with default parameters of fixed penalty motif criteria ([Kofler, Schlotterer, & Lelley, 2007](#_ENREF_5)). We analyzed the counts, average length and relative abundance of different motif categories to characterize the distribution of microsatellites in the *C. vestalis* genome.

The primers were designed using PRIMER 3 implemented in the software MSATCOMMANDER 1.0.8 ([Faircloth, 2008](#_ENREF_3)) with the following criteria: (i) GC content 45-55%; (ii) primer length 19-22 bp (20 optimum); (iii) melting temperature 55-62 °C (60 °C optimum); and (iv) product size 150-500 bp.

**Primer validation and evaluation**

Seventy primer pairs were chosen and synthesized to test for polymorphism and amplification efficiency. To improve efficiency, we added M13 primer (TGTAAAACGACGGCCAGT) to the 5’ end of forward primers ([Schuelke, 2000](#_ENREF_7)). PCR amplifications were performed in Mastercycler pro (Eppendorf, Germany, Hamburg) in 10 μl reactions containing 0.5 μl of genomic DNA (5-20 ng/μl), 1 μl of reaction buffer, 1 μl of MgCl_2_, 1.6 μl of dNTPs, 0.04 μl of forward primer (10 mM), 0.16 μl of reverse primer (10 mM), 0.32 μl M13 primer (10 mM) labeled with fluorescence and 0.1 μl *Taq* DNA polymerase using the following temperature condition: 95 °C for 10 min; 30 cycles at 95 °C for 30 s, 58 °C or 53 °C for 45 s, 72 °C for 45 s; 8 cycles at 95 °C for 30 s, 53 °C for 45 s, 72 °C for 45 s; and a final extension step at 72 °C for 10 min. The size of the amplified PCR products was determined using an ABI 3730xl DNA Analyzer (Applied Biosystems, Foster, CA, USA) with GeneScan 500 LIZ size standard (Applied Biosystems). Allele designation was obtained using the software GENEMAPPER v4.0 (Applied Biosystems). Primers that exhibited amplification > 75% and polymorphisms were further characterized using 40 *C. vestalis* individuals.

**Results**

Seventeen microsatellite loci were chosen for further studies of *C. vestalis* after validation (Table S1).

**Table S1** Characteristics of 17 microsatellite loci developed for *Cotesia* *vestalis*.

| Locus | Scaffold | Motif | Forward primer | Reverse primer | Tm | Product size (bp) |
| --- | --- | --- | --- | --- | --- | --- |
| CV-S01 | Scaffold83 | ATC(9) | TCGTACCACTTTAACTAACGCC | GGTTTAACTTCCACTCGCGG | 58.00 | 226 |
| CV-S06 | Scaffold173 | AC(10) | ACAAATGAGGAGCCAGTGTG | AAACCTGACAATATGGGCGC | 53.00 | 269 |
| CV-S12 | Scaffold715 | AAT(10) | CTCTGTGGTTCGAGGCAAAC | ATTTACTCACAACGTCCGGC | 58.00 | 264 |
| CV-S18 | Scaffold29 | AAGAG(4) | TCCACGTCTATGACTTCGGC | TCCTGCCAGACGAATGACTC | 58.00 | 192 |
| CV-S19 | Scaffold132 | AC(10) | TCACCGGCGTTTAATCAGTC | TCAACAACCTCTCCTCCAGC | 58.00 | 302 |
| CV-S25 | Scaffold72 | AC(20) | ACCGAGCAATTTGAACACAG | CAACCTCGCTTTGATCCGTC | 58.00 | 280 |
| CV-S28 | Scaffold54 | AT(15) | AGGGTGAATTCAGAGAGCAC | CTACCTTATACATCGCGCCG | 58.00 | 198 |
| CV-S32 | Scaffold326 | AAC(9) | AAGACATGATGCGGCGTTAG | GTGGCTATGCGATCTCCTTTG | 58.00 | 323 |
| CV-S34 | Scaffold15 | AAG(11) | CGGGCAATTAGTTTCTCCGTC | TTTCCACCGCTCCTGGTATC | 58.00 | 331 |
| CV-S38 | Scaffold29 | AAG(34) | AGAGAACCAAGAGCAAGACC | ACAAATGATCTCGGTTGCGC | 58.00 | 268 |
| CV-S42 | Scaffold15 | AG(19) | GCCAATCTCGTCTTCAGGTG | AGCGGTTGACTATGCACCAG | 58.00 | 340 |
| CV-S46 | Scaffold79 | AGC(11) | ACGCAATCACCAAGCATCAC | ATGCGGACGGAATAGAGACG | 58.00 | 247 |
| CV-S59 | Scaffold1 | AG(11) | ATCTGGTTTCGTGTGTCTCG | TTGCTCTACTCTGGCGACTC | 58.00 | 329 |
| CV-S60 | Scaffold15 | AAAAT(6) | ACACAGTTAACTACACCCGC | TTCCTCCTGTTGCTCCGAAG | 58.00 | 268 |
| CV-S61 | - | (AT)10 | AATTGGTTCTCTCGTAGCGG | ATCCCTTCGATCCCATCACG | 58.00 | 188 |
| CV-S66 | Scaffold459 | ATC(13) | AAGAAGCCACGCATCAACAG | TTGAGACCTAAAGACCCGCG | 58.00 | 322 |
| CV-S69 | Scaffold88 | AAC(9) | AGCAGCTCAGTTACGCAATG | CATTCATCAGCATGTCCGGC | 58.00 | 161 |

Tm, annealing temperature.

**Appendix S2** Bottleneck analysis and effective population size estimation of *Cotesia vestalis* and *Plutella xylostella*

We tested for recent bottlenecks using the heterozygote excess method in Bottleneck 1.2.02 ([Piry, Luikart, & Cornuet, 1999](#_ENREF_6)). We examined the infinite alleles model (IAM), step-wise mutation model (SMM), and the hybrid two-phase model (TPM). Bottlenecks were found in the JL population of *C. vestalis* and GD population of *P. xylostella* under most models. Results were shown in Table S2.

Effective population size of each population in *Cotesia vestalis* and DBM was estimated using the sibship frequency (SF) method as implemented in COLONY ([Jones & Wang, 2010](#_ENREF_4)) and linkage disequilibrium (LD), heterozygote excess (HE) and molecular coancestry (MC) methods as implemented in NEESTIMATOR version 2.01 ([Do et al., 2014](#_ENREF_2)). Although values estimated using different methods varied largely, the effective population sizes of *C. vestalis* and DBM are similar when estimated by the same method (Table A). Simulation study showed that the SF method is more accurate and suitable for haplodiploid species and is more robust to the presence of linkage and genotyping errors of markers than the other methods ([Wang, 2016](#_ENREF_8)). When the SF was used, estimated values of effective population size for both species are around 100, except for the JL population with a relative small value, and the X1 and SC populations with very large values. The small value might be caused by the collection of siblings while the large values might be caused by the small number of individuals used in the population. Several values estimated by LD and all values estimated by HE are infinite. Values of *C. vestalis* estimated by MC ranged from 4.0 (JL) to 340.8 (SC), while that of DBM ranged from 7.1 to 63.8, except for the Yunnan population (YN) whose population was estimated to be infinite (Table S3).

**Table S2** P-values from 1-sided signed-rank tests for heterozygote excess. Values under the TMP models indicate the variance in TPM and proportion of SMM in TPM (as a %).

| Species | Population | IAM | SMM | TPM  (30, 10) | TPM  (30, 30) | TPM  (30, 60) | TPM  (30, 80) | TPM  (15, 10) | TPM  (15, 30) | TPM  (15, 60) | TPM  (15, 80) |
| --- | --- | --- | --- | --- | --- | --- | --- | --- | --- | --- | --- |
| *Cotesia vestalis* | HJ | 0.0020 | 0.2402 | 0.0119 | 0.0119 | 0.0103 | 0.0459 | 0.0101 | 0.0114 | 0.0118 | 0.0450 |
|  | JL | 0.0001 | 0.1051 | 0.0002 | 0.0016 | 0.0013 | 0.0017 | 0.0001 | 0.0014 | 0.0013 | 0.0110 |
|  | SX | 0.1233 | 0.2020 | 0.1112 | 0.2723 | 0.4505 | 0.5743 | 0.2868 | 0.2727 | 0.4391 | 0.5349 |
|  | BJ | 0.0092 | 0.4027 | 0.0378 | 0.0381 | 0.0371 | 0.0401 | 0.0393 | 0.0392 | 0.0368 | 0.0408 |
|  | SD | 0.4352 | 0.0923 | 0.5235 | 0.5278 | 0.4395 | 0.2355 | 0.5267 | 0.5568 | 0.5833 | 0.2196 |
|  | JH | 0.2491 | 0.1096 | 0.2578 | 0.2515 | 0.4199 | 0.5489 | 0.2720 | 0.2529 | 0.4273 | 0.3632 |
|  | GD | 0.5635 | 0.0032 | 0.2139 | 0.1920 | 0.1032 | 0.0950 | 0.2132 | 0.2127 | 0.0992 | 0.0402 |
|  | GX | 0.1084 | 0.0033 | 0.4130 | 0.4302 | 0.5637 | 0.3665 | 0.4227 | 0.5710 | 0.3729 | 0.2139 |
|  | YN | 0.2599 | 0.0001 | 0.3739 | 0.3831 | 0.1055 | 0.0949 | 0.3729 | 0.3693 | 0.1006 | 0.0935 |
|  | SC | 0.2982 | 0.0644 | 0.4920 | 0.4671 | 0.2649 | 0.2007 | 0.4818 | 0.3203 | 0.2431 | 0.1859 |
|  | X2 | 0.2356 | 0.0375 | 0.4385 | 0.5850 | 0.5629 | 0.3768 | 0.5700 | 0.5758 | 0.5733 | 0.3770 |
|  | X1 | 0.3942 | 0.1197 | 0.5388 | 0.3505 | 0.3826 | 0.3028 | 0.3938 | 0.3653 | 0.3388 | 0.2781 |
| *Plutella xylostella* | BJ | 0.0764 | 0.0283 | 0.2538 | 0.2485 | 0.5038 | 0.5316 | 0.2499 | 0.2338 | 0.4748 | 0.5377 |
|  | GD | 0.4781 | 0.0048 | 0.0265 | 0.0035 | 0.0039 | 0.0046 | 0.0033 | 0.0042 | 0.0039 | 0.0040 |
|  | JL | 0.2570 | 0.0263 | 0.2391 | 0.5041 | 0.4726 | 0.5155 | 0.5194 | 0.5247 | 0.5107 | 0.0998 |
|  | JH | 0.4940 | 0.1051 | 0.4982 | 0.4980 | 0.4715 | 0.4690 | 0.4773 | 0.4804 | 0.4734 | 0.4648 |
|  | X2 | 0.2253 | 0.2536 | 0.5091 | 0.4765 | 0.5244 | 0.5178 | 0.5116 | 0.5079 | 0.5015 | 0.5267 |
|  | YN | 0.2578 | 0.0309 | 0.2634 | 0.2580 | 0.2716 | 0.2758 | 0.2607 | 0.2656 | 0.2690 | 0.2626 |

**Table S3** Values of estimated effective populations sizes based on four methods

| Species | Population | SF (random mating) | SF (nonrandom mating) | LD | HE | MC |
| --- | --- | --- | --- | --- | --- | --- |
| *Cotesia*  *vestalis* | X1 | 2147483647 | 2147483647 | Infinite | Infinite | 21.0 |
|  | X2 | 102 | 74 | 597.7 | Infinite | 23.3 |
|  | HJ | 59 | 54 | 22.8 | Infinite | 34.4 |
|  | JL | 10 | 9 | 1.8 | Infinite | 4.0 |
|  | BJ | 39 | 37 | 37.4 | Infinite | 13.8 |
|  | SD | 81 | 92 | 148.0 | Infinite | 11.6 |
|  | SX | 29 | 34 | 211.0 | Infinite | 15.2 |
|  | SC | 2147483647 | 2147483647 | Infinite | Infinite | 340.8 |
|  | JH | 149 | 140 | Infinite | Infinite | 16.0 |
|  | GD | 39 | 43 | 36.3 | Infinite | 8.6 |
|  | GX | 115 | 100 | 127.1 | Infinite | 7.8 |
|  | YN | 143 | 138 | Infinite | Infinite | 17.0 |
| *Plutella*  *xylostella* | X1 | 420 | 244 | Infinite | Infinite | 33.1 |
|  | JL | 92 | 54 | 313.7 | Infinite | 63.8 |
|  | BJ | 158 | 84 | 24127.1 | Infinite | 7.1 |
|  | JH | 92 | 52 | 1464.9 | Infinite | 16.7 |
|  | GD | 85 | 51 | 110.8 | Infinite | 10.3 |
|  | YN | 32 | 22 | 39.0 | Infinite | Infinite |

SF, sibship frequency; LD, linkage disequilibrium; HE, heterozygote excess; MC, molecular coancestry.

**Appendix S3** BAPS analysis of the population genetic structure of *Cotesia vestalis*

A spatially explicit BAPS model for clustering of individuals implemented in BAPS version 6.0 ([Corander et al., 2004](#_ENREF_1)) was used for microsatellite loci and mitochondrial genes of *C. vestalis*. This model combines sample locations with the likelihood of genetic groupings and is particularly efficient with large data sets. As the *K* values (maximum number of genetically diverged groups) affect the initial assignment in simulations, the possibility of finding only a local mode is reduced when the simulation is started many times from different initial assignments; we therefore performed 20 runs (*K* = 20) for mitochondrial genes and 20 runs (*K* = 5) for microsatellite loci to ensure convergence and consistency of the results.

BAPS analysis of mitochondrial genes in *C. vestalis* divided the 12 populations into 3 clusters. The first cluster was composed of individuals mainly from four locations from northern China. The second cluster was composed of individuals mainly from YN and a few individuals from GX in southwestern China. The third cluster was the largest one, which was mainly distributed in the central, eastern and southern populations, and also found in the other populations except for X1 and X2. The microsattelite analysis also showed four groupings, but in this case the eastern populations separated out along with YN from the main group (Fig. S1).


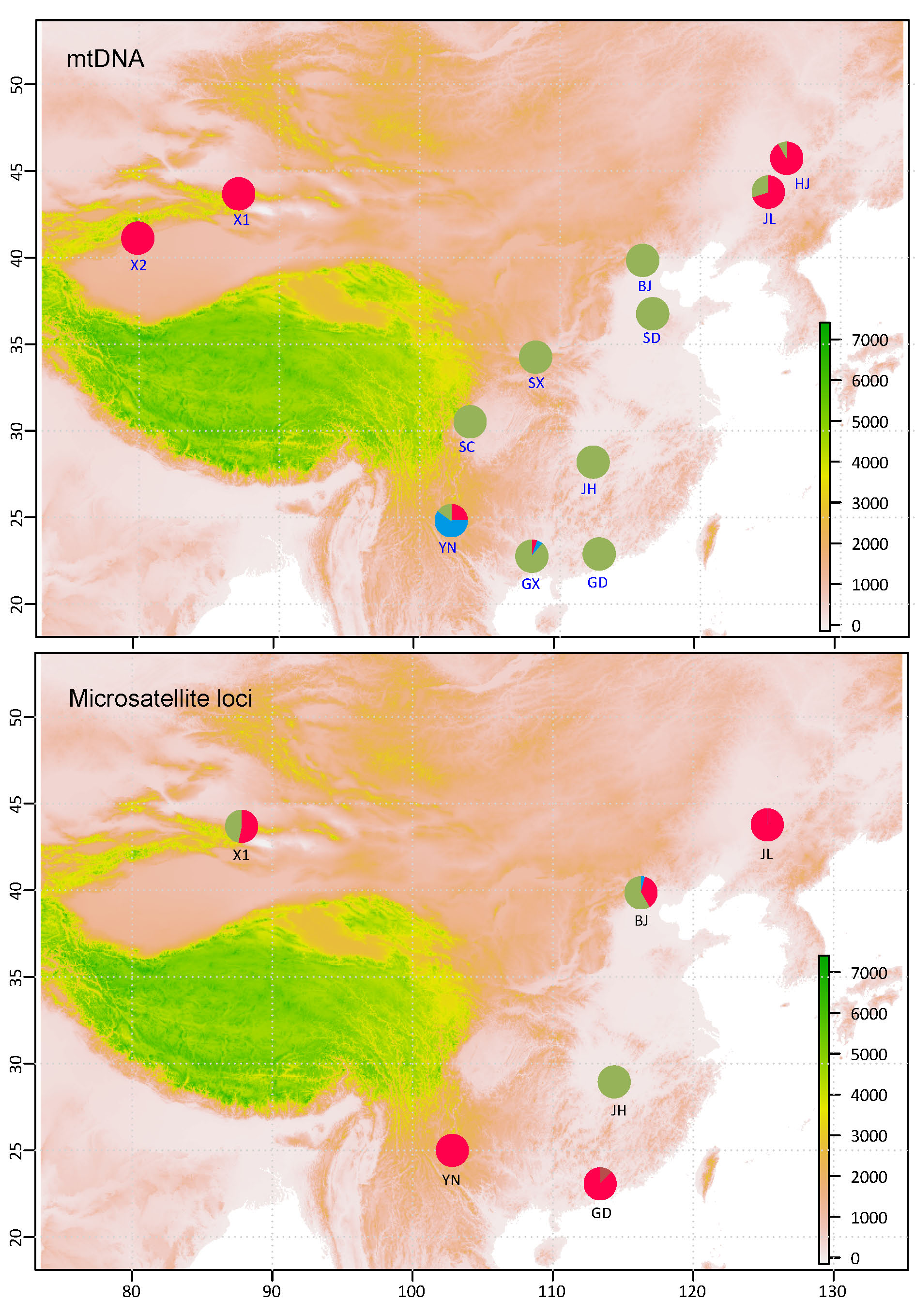


**Fig. S1** Results of BAPS analysis of *Cotesia vestalis* based on mitochondrial genes (top) and microsatellite loci (bottom). The areas of different colors in each population correspond to the proportion of each cluster in the population. Latitudes (N, y axis) and longitudes (E, x axis) are given.

**References**

Corander, J., Waldmann, P., Marttinen, P., & Sillanpaa, M. J. (2004). BAPS 2: enhanced possibilities for the analysis of genetic population structure. *Bioinformatics*, *20*, 2363-9.

Do, C., Waples, R. S., Peel, D., Macbeth, G. M., Tillett, B. J., & Ovenden, J. R. (2014). NeEstimator v2: re-implementation of software for the estimation of contemporary effective population size (Ne ) from genetic data. *Mol Ecol Resour*, *14*, 209-14.

Faircloth, B. C. (2008). msatcommander: detection of microsatellite repeat arrays and automated, locus-specific primer design. *Molecular Ecology Resources*, *8*, 92-4.

Jones, O. R., & Wang, J. (2010). COLONY: a program for parentage and sibship inference from multilocus genotype data. *Mol Ecol Resour*, *10*, 551-5.

Kofler, R., Schlotterer, C., & Lelley, T. (2007). SciRoKo: a new tool for whole genome microsatellite search and investigation. *Bioinformatics*, *23*, 1683-5.

Piry, Sylvain, Luikart, Gordon, & Cornuet, Jean-Marie. (1999). BOTTLENECK: a program for detecting recent effective population size reductions from allele data frequencies. *Journal of Heredity*, *90*, 502-503.

Schuelke, M. (2000). An economic method for the fluorescent labeling of PCR fragments. *Nature Biotechnology*, *18*, 233-4.

Wang, J. (2016). A comparison of single-sample estimators of effective population sizes from genetic marker data. *Molecular Ecology*, *25*, 4692-711.
